# Competitive amplification of differentially melting amplicons (CADMA) improves *KRAS* hotspot mutation testing in colorectal cancer

**DOI:** 10.1186/1471-2407-12-548

**Published:** 2012-11-23

**Authors:** Lasse Sommer Kristensen, Tina Ellegaard Kjeldsen, Henrik Hager, Lise Lotte Hansen

**Affiliations:** 1 Department of Biomedicine, University of Aarhus, Bartholin Building, Wilhelm Meyers Allé 4, DK-8000, Aarhus C, Denmark; 2 Department of Pathology, Aarhus University Hospital, Nørrebrogade 45, DK-8000, Aarhus C, Denmark

**Keywords:** Colorectal Cancer, *EGFR*, *KRAS*, DNA mutational analyses, CADMA

## Abstract

**Background:**

Cancer is an extremely heterogeneous group of diseases traditionally categorized according to tissue of origin. However, even among patients with the same cancer subtype the cellular alterations at the molecular level are often very different. Several new therapies targeting specific molecular changes found in individual patients have initiated the era of personalized therapy and significantly improved patient care. In metastatic colorectal cancer (mCRC) a selected group of patients with wild-type *KRAS* respond to antibodies against the epidermal growth factor receptor (EGFR). Testing for *KRAS* mutations is now required prior to anti-EGFR treatment, however, less sensitive methods based on conventional PCR regularly fail to detect *KRAS* mutations in clinical samples.

**Methods:**

We have developed sensitive and specific assays for detection of the seven most common *KRAS* mutations based on a novel methodology named Competitive Amplification of Differentially Melting Amplicons (CADMA). The clinical applicability of these assays was assessed by analyzing 100 colorectal cancer samples, for which *KRAS* mutation status has been evaluated by the commercially available TheraScreen® KRAS mutation kit.

**Results:**

The CADMA assays were sensitive to at least 0.5% mutant alleles in a wild-type background when using 50 nanograms of DNA in the reactions. Consensus between CADMA and the TheraScreen kit was observed in 96% of the colorectal cancer samples. In cases where disagreement was observed the CADMA result could be confirmed by a previously published assay based on TaqMan probes and by *fast* COLD-PCR followed by Sanger sequencing.

**Conclusions:**

The high analytical sensitivity and specificity of CADMA may increase diagnostic sensitivity and specificity of *KRAS* mutation testing in mCRC patients.

## Background

Cancer is the result of a somatic microevolution in which cells acquire specific growth advantages through a stepwise accumulation of mitotically heritable changes in the function of cancer related genes, which may be oncogenes, tumor suppressor genes, and DNA repair genes. Most cancers are extremely complex at the molecular level, and two patients suffering from the same cancer disease may have acquired very different cellular alterations. Consequently, there is a huge potential in individualizing patient treatment using molecular biomarkers as predictors for response or non-response to targeted therapy.

New therapies targeting specific molecular changes in tumors of individual patients have started to introduce a paradigm shift for cancer treatment. Most cancer patients are now tested for one or more molecular biomarkers in order to determine optimal treatment strategies for the individual patient [1].

Activating somatic mutations in the *KRAS* oncogene is an example of a biomarker, which predicts non-response to therapies targeting the epidermal growth factor receptor (EGFR) in metastatic Colorectal Cancer (mCRC) [2,3]. EGFR and KRAS are part of the same signaling pathway, and EGFR overexpression as well as activating *KRAS* mutations contribute to development and progression of several human cancers, including CRC. An important feature of mutant KRAS is its ability to transmit growth promoting signals independent of EGFR activation. This is the biological explanation why anti-EGFR treatment fails to inhibit progression of *KRAS* mutated tumors. Activating mutations in *KRAS* are most often found in a mutation hotspot comprising codon 12 and 13 of exon 2. Therefore, the U.S. Food and Drug Administration (FDA) and the European Medicines Agency (EMA) require that patients are tested for *KRAS* hotspot mutations prior to anti-EGFR treatment using the approved drugs, panitumumab and cetuximab. However, evidence is present that patients harboring the codon 13 c.38 G > A mutation may benefit from anti-EGFR treatment [4,5].

We have recently evaluated several methods for the detection of *KRAS* mutations in clinical samples. The frequency of mutated samples was found to be influenced by the analytical sensitivity of the method applied [6]. In particular, conventional PCR followed by high-resolution melting (HRM) or sequencing failed to detect mutations in a substantial number of samples due to the limited sensitivity of this approach. This may, in part, be caused by intra tumor heterogeneity and contamination with wild-type DNA from normal cells, which typically are observed in infiltrating cancers. We also evaluated a commercially available kit, the TheraScreen® *KRAS* mutation kit (QIAGEN, Hilden, Germany), and an assay based on *fast* COLD-PCR, which enriches for mutant sequences by using a lower denaturation temperature in the PCR [7]. These methods are more sensitive, and were capable of identifying additional mutated samples not detected by conventional PCR. However, *fast* COLD-PCR failed to increase the sensitivity of melting temperature retaining mutations, and the TheraScreen kit is more time-consuming and less cost-effective compared to HRM followed by sequencing of positive samples [8].

Therefore, we have developed a new method, Competitive Amplification of Differentially Melting Amplicons (CADMA), which enables very sensitive mutation detection regardless of the melting properties of the mutations to be detected [9].

In this contribution, we have designed and optimized CADMA assays for the seven most common *KRAS* exon 2 hotspot mutations. The sensitivity and specificity of each assay were evaluated using serial dilutions of cell line DNA containing the relevant mutations in a wild-type background. We further evaluated the potential of these assays for the detection of *KRAS* mutations in CRC samples derived from formalin fixed paraffin embedded (FFPE) tissues. In total, we have tested 100 samples using the CADMA assays, and compared these results with results obtained using the TheraScreen® *KRAS* mutation kit, which tests for the same seven mutations. Samples, which did not give the same result by CADMA and the TheraScreen kit, were tested using a previously published highly sensitive TaqMan based assay [10] and by *fast* COLD-PCR followed by Sanger sequencing.

## Methods

### Samples and DNA extraction

Formalin-Fixed Paraffin Embedded (FFPE) blocks from surgical biopsies from 100 patients diagnosed with adenocarcinoma in colon were selected from the archives at the Department of Pathology, Aarhus University Hospital. The specimens were up to 10 years old. For each sample, six tissue sections of 10 μm were used for DNA extraction. Deparaffinization and DNA extraction were performed as previously described [6].

DNA from peripheral blood (PB) obtained from medical students were used as wild-type controls. The DNA was extracted following a modified salt precipitation protocol as previously described [11]. The DNA was taken from a biobank for which informed consent was provided for each sample. The approval by the regional ethics comity has the journal number 2001–2.0/37.

The Local Ethical Committee, Aarhus County, Denmark, approved this study.

### Cell lines and dilution series

Seven different cell lines each containing different *KRAS* mutations were used in this study; A549 (c.34 G > A, codon 12), DLD-1 (c.38 G > A, codon 13), LS174T (c.35 G > A, codon 12), NCI-H23 (c.34 G > T, codon 12) PSN-1 (c.34 G > C, codon 12), RPMI 8226 (c.35 G > C, codon 12), and SW480 (c.35 G > T, codon 12). The cell lines were cultured and harvested, and the DNA was extracted as described [6], with the exception of PSN-1 for which extracted DNA was purchased from Health Protection Agency Culture Collection, UK, and NCI-H23 for which extracted DNA was kindly donated by Professor Dmitri Loukinov, NIAID/NIH.

DNA from each cell line was quantified using a NanoDrop ND-1000 spectrophotometer (NanoDrop Technologies, Wilmington, DE) and serially diluted into wild-type DNA to the following fractions of mutated alleles in a wild-type background; 50%, 10%, 1%, and 0.5% (assuming no pipetting errors and that all cell lines are monoclonal).

### CADMA primer design

The primer sequences were designed to target the *KRAS* sequence obtained from GenBank http://www.ncbi.nlm.nih.gov/GenBank/ (*KRAS* GenBank accession number NM_033360.2). Mutation specific primers were designed for the seven most common *KRAS* exon 2 mutations. These primers each introduce two melting temperature decreasing mutations. The overlapping primer and the common primer were designed to avoid pseudogene amplification and were the same for all assays. The primers for detection of the c.35 G > C mutation has been published previously [9]. Primer sequences can be found in Table 1.

**Table 1:** Details of the CADMA assays

**Mutation**	**Cell line**	**Primers (introduced mutations are underlined)**	**Primer concentrations**	**Annealing temperature**
c.34 G > A	A549	Mutation specific forward:	400 nM	57°C
		GAATATAAACTTATGGTAGTTGGAGATA		
		Overlapping forward:	100 nM	
		ATGACTGAATATAAACTTGTGGTAGTTG		
		Common reverse:	400 nM	
		ACTGTCAAGGCACTCTTGCCTAC		
c.34 G > T	NCI-H23	Mutation specific forward:	400 nM	60°C
		GAATATAAACTTGTAGTAATTGGAGCTT		
		Overlapping forward:	150 nM	
		ATGACTGAATATAAACTTGTGGTAGTTG		
		Common reverse:	400 nM	
		ACTGTCAAGGCACTCTTGCCTAC		
c.34 G > C	PSN1	Mutation specific forward:	400 nM	60°C
		GAATATAAACTTGTAGTAATTGGAGCTC		
		Overlapping forward:	100 nM	
		ATGACTGAATATAAACTTGTGGTAGTTG		
		Common reverse:	400 nM	
		ACTGTCAAGGCACTCTTGCCTAC		
c.35 G > A	LS174T	Mutation specific forward:	400 nM	60°C
		GAATATAAACTTGTGGTAATTGGAGATGA		
		Overlapping forward:	150 nM	
		ATGACTGAATATAAACTTGTGGTAGTTG		
		Common reverse:	400 nM	
		ACTGTCAAGGCACTCTTGCCTAC		
c.35 G > T	SW480	Mutation specific forward:	400 nM	64°C
		GAATATAAACTTGTAGTAATTGGAGCTGT		
		Overlapping forward:	150 nM	
		ATGACTGAATATAAACTTGTGGTAGTTG		
		Common reverse:	400 nM	
		ACTGTCAAGGCACTCTTGCCTAC		
c.35 G > C	RPMI8226	Mutation specific forward:	400 nM	58°C
		GAATATAAACTTGTAGTAATTGGAGCTGC		
		Overlapping forward:	100 nM	
		ATGACTGAATATAAACTTGTGGTAGTTG		
		Common reverse:	400 nM	
		ACTGTCAAGGCACTCTTGCCTAC		
c.38 G > A	DLD-1	Mutation specific forward:	400 nM	62°C
		AAACTTGTGGTAGTTGGAGATGGTTA		
		Overlapping forward:	150 nM	
		ATGACTGAATATAAACTTGTGGTAGTTG		
		Common reverse:	400 nM	
		ACTGTCAAGGCACTCTTGCCTAC		

Primer sequences are given in 5’ → 3’ directions.

### PCR and HRM Conditions for the CADMA assays

PCR cycling and HRM analysis were performed on the Rotor-Gene 6000™ (Corbett Research, Sydney, Australia) or the Rotorgene Q (Qiagen, Hilden, Germany). SYTO® 9 (Invitrogen, Eugene, USA) was used as intercalating dye. The final reaction mixtures consisted of 50 ng of DNA, 1x PCR buffer, 2.5 mmol/L MgCl_2_, optimized relative primer concentrations (Table 1), 200 μmol/L of each dNTP, 5 μmol/L of SYTO® 9, 0.5U of HotStarTaq (Qiagen) (5U/μL) in a volume of 20 μL. The CADMA cycling protocol was initiated by one cycle at 95°C for 15 min, followed by 45 cycles of 95°C for 10 s, annealing temperature (T_A_) for 20 s (Table 1), 72°C for 20 s, and one cycle at 95°C for 1 min. HRM was performed from 65°C to 95°C with a temperature increase of 0.1°C/s. Samples were analyzed in triplicates (cell line experiments) or in duplicates (colorectal cancer specimens).

### CADMA data analysis

The Rotorgene 6000 Series Software version 1.7.87 supplied with the instrument was used to analyze the data. When analyzing the mCRC samples, standards containing 1% mutant alleles were used as cut-off point to facilitate direct comparison with the TheraScreen® kit. The samples were scored manually by visual inspection of the derivative of the raw data (melt curve analysis) and the normalized HRM and difference graphs (high resolution melting analysis). For the difference graphs a wild-type sample was selected as reference. The samples were tested with each of seven CADMA assays, however, when a sample was found to be mutation positive it was not tested using the remaining CADMA assays unless the result was in disagreement with the result provided by the TheraScreen kit.

### Mutation analysis using the TheraScreen® *KRAS* mutation Kit

The mCRC samples were analyzed using the TheraScreen® *KRAS* mutation kit (Qiagen). This kit analyzes the mutation status for the seven most commonly found *KRAS* exon 2 mutations by a technology that combines ARMS® (allele specific PCR) with Scorpions® real-time PCR. The manufacturer has reported the sensitivity to be 1% mutant alleles in a wild-type background if sufficient DNA input is used.

### Alelle-specific PCR (TaqMan)

The TaqMan based allele-specific PCR assay used herein has been published recently by Lang et al. [10]. We used the same PCR conditions and real-time PCR instrument as described. This assay determines mutation status using a predetermined cutoff ΔCt value (Ct [allele-specific assay] – Ct [reference assay]) as described [10]. The analytical sensitivity of the assays were reported to be 1% mutant alleles in a wild-type background [10]. Samples were analyzed in duplicates for all TaqMan experiments and the average Ct value of the duplicates was used to calculate ΔCt values.

### COLD-PCR followed by Sanger sequencing

The COLD-PCR was essentially performed as previously described [6]. However, only 10 standard PCR cycles were performed prior to the COLD-PCR cycles using a critical temperature of 78°C. Sanger sequencing was performed using M13 tagged primers to create a longer amplicon, which could be successfully sequenced as previously described [6]. The COLD-PCR assay could detect 0.5% DNA from the A549 cell line diluted in wild-type DNA, and 1% DNA from the LS174T cell line diluted in wild-type DNA (data not shown).

## Results

### The analytical sensitivity of the CADMA assays for the detection of *KRAS* hotspot mutations

**Figure 1:**
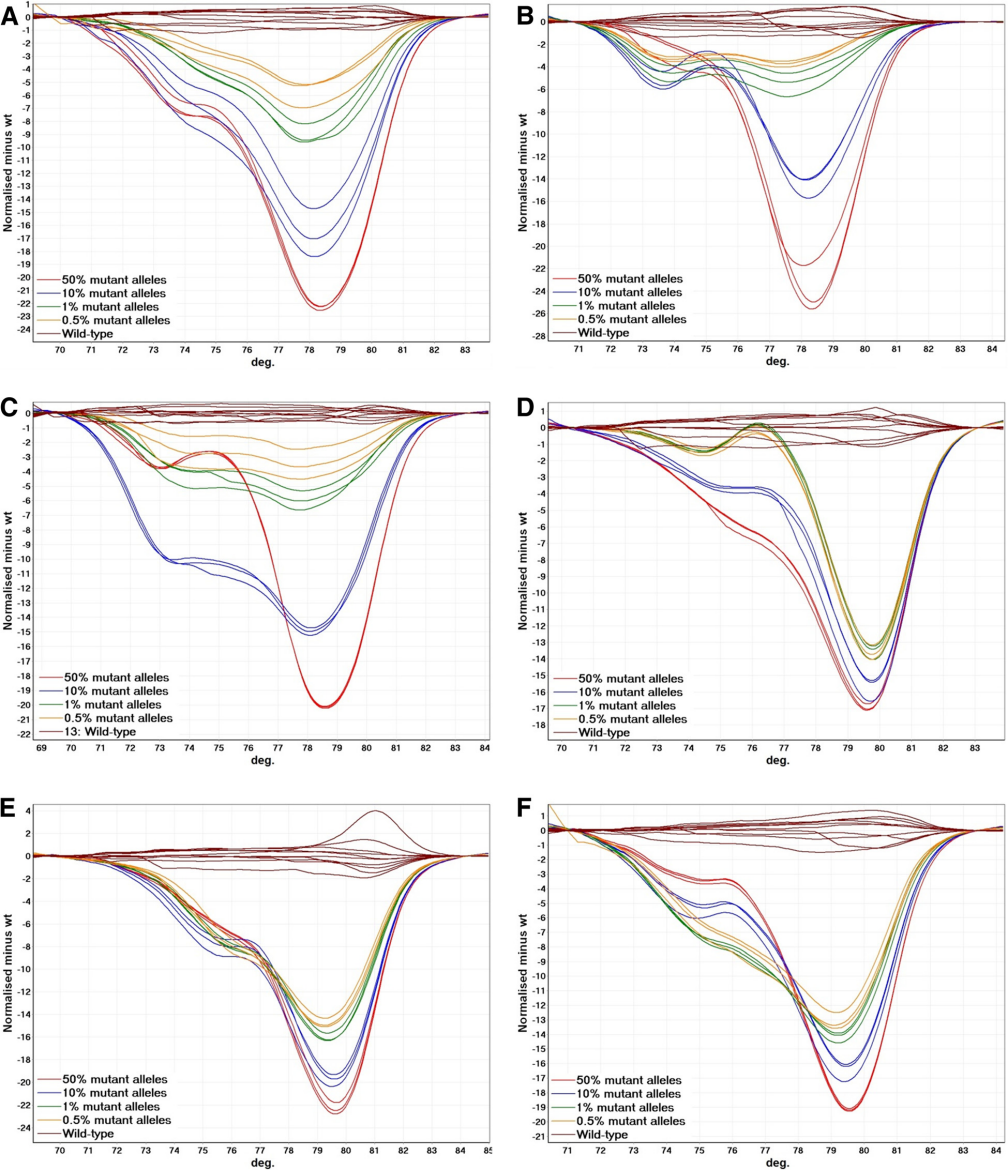
**The analytical sensitivity and specificity of the CADMA assays performed using the Rotorgene 6000.** Ten wild-type replicates were run together with a standard dilution series of mutant alleles from cell lines carrying the relevant mutations in a wild-type background (50%, 10%, 1%, and 0.5%) in triplicates. The three replicates of the 0.5% standard could all be distinguished from ten wild-type replicates in all assays. **A**. The c.34 G > A CADMA assay. **B**. The c.38 G > A CADMA assay. **C**. The c.35 G > A CADMA assay. **D**. The c.34 G > C CADMA assay. **E**. The c.35 G > T CADMA assay. **F**. The c.34 G > T CADMA assay.

The CADMA assays were optimized to avoid false amplification from wild-type sequences by the mutation specific primer, while maintaining a high sensitivity as previously described [9]. Hereafter, the sensitivity and specificity was evaluated by analyzing ten wild-type replicates together with a standard dilution series of mutant alleles into wild-type alleles (50%, 10%, 1%, and 0.5%) in triplicates. All three replicates of the 0.5% standard could be distinguished from all ten wild-type replicates in all assays (Figure 1).

To assess in between run variation, we repeated each of these experiments using the Rotorgene Q (Qiagen, Hilden, Germany) in a different laboratory. Again, the three replicates of the 0.5% standard could all be distinguished from ten wild-type replicates in all assays, and no false amplification in wild-type reactions was observed (Additional file 1: Figure S1).

### *KRAS* hotspot mutation analysis in CRC samples using CADMA and the TheraScreen® *KRAS* mutation Kit

One-hundred mCRC samples derived from FFPE tissues were analyzed for *KRAS* mutations using the CADMA assays and the TheraScreen® kit. One and two samples failed to amplify, when using CADMA and the TheraScreen® kit, respectively. These samples were scored as “no data”. Using the TheraScreen kit 45/98 (45.9%) of the samples were found to carry a *KRAS* exon 2 mutation. Using CADMA 44/99 (44.4%) of the samples were mutation positive. Consensus between the two methods was found in 93/97 (95.9%) of the samples. The results are summarized in Additional file 2: Table S1.

**Figure 2:**
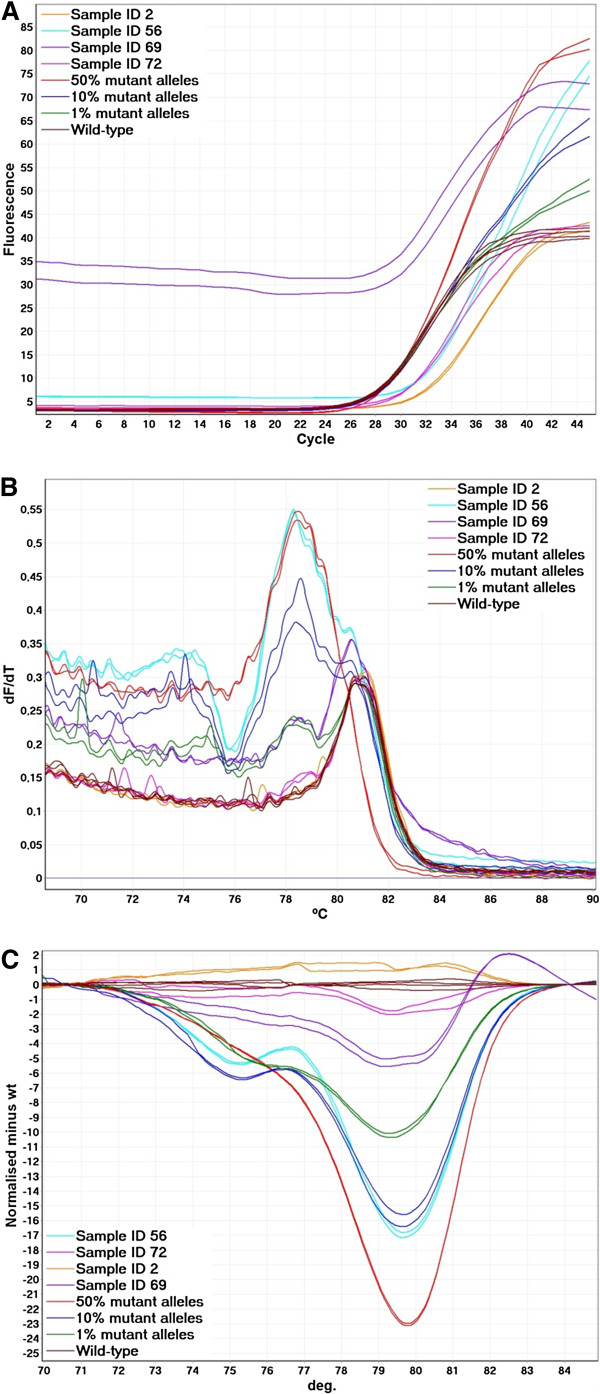
**Examples from screening of mCRC samples using the c.34 G > T CADMA assay. A**. Real-time amplification data. High background fluorescence can be observed for sample ID 69. **B**. The derivative of the raw melting data (melt curve analysis). Sample ID 56 carry the c.34 G > T mutation. Sample ID 2, 69 and 72 were negative for the c.34 G > T mutation. Sample ID 69 and 72 may carry another *KRAS* mutation as small deviations from the wild-type replicates can be observed. Sample ID 69, which gave high fluorescence during the PCR amplification, has deviating melt curves from 82 to 88°C. **C**. Normalized HRM difference graph. Of the mCRC samples shown only one (sample ID 56) deviates more from the wild-type replicates than the standard containing 1% mutant alleles.

Examples from the c.34 G > T CADMA assay are shown in Figure 2. Since the overlapping CADMA primer amplifies both mutated and wild-type sequences each CADMA assay may detect *KRAS* mutations other than the one targeted by the mutation specific primer albeit at a lower sensitivity. The shape of the melting curves could easily be used to distinguish the mutation, targeted by each CADMA assay, from other mutations detected by the overlapping primer, as the resulting amplicons have different melting properties, due to the two additional mutations incorporated by the mutation specific primer. Examples of this are shown in Figure 2. When samples amplify late this may cause the melting curves to be shifted [12], and other abnormalities, such as the one shown for sample ID 69 in Figure 2, may also result in deviations of the melting curves. Shifted melting curves may result in differences in the normalized HRM and difference graphs, which could lead to wrong interpretation of the results, if the melting curves are not inspected.

**Figure 3:**
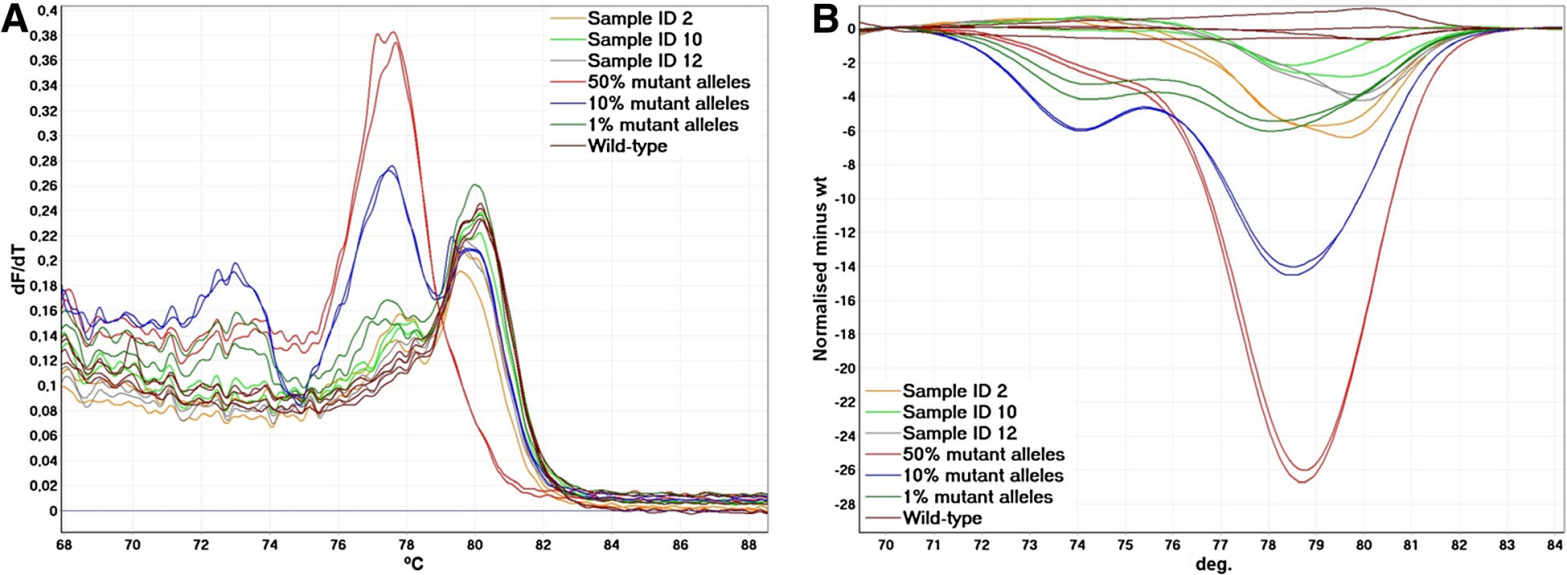
**Examples from screening of mCRC samples using the c.38 G > T CADMA assay.** The wild-type mCRC samples showed more variation in the c.38 G > A CADMA assay compared to any of the other CADMA assays. **A**. The derivative of the raw melting data (melt curve analysis). No heteroduplexes, which melt between 71 and 74°C, can be observed in any of the wild-type mCRC samples. **B**. Normalized HRM difference graph. Sample ID 2 deviates more from the wild-type replicates than the standard containing 1% mutant alleles from approximately 79 to 81°C. This should not be interpreted as a c.38 G > T mutation, since no heteroduplexes are present.

When testing DNA samples derived from FFPE tissues more variation in the melt curves is likely to be observed compared to DNA samples of high quality [9]. For this reason, it is also important to analyze the samples of unknown mutation status relative to standards of known ratios of wild-type to mutant alleles. Generally, the wild-type samples showed more variation in the c.38 G > A CADMA assay compared to any of the other CADMA assays (Figure 3). However, this did not give rise to misclassification of any of the samples, and the wild-type status of sample ID 10 and 12 shown in Figure 3 could be confirmed by COLD-PCR followed by sequencing (data not shown). However, sample ID 2 was shown to contain a c.37 G > A and a c.39 C > G mutation, which are not tested for by the TheraScreen kit and CADMA. These mutations are likely to be present at very low levels as they were found only when sequencing in the forward direction, or alternatively they may be sequencing errors.

### Confirmation of the CADMA results by TaqMan and by COLD-PCR followed by sequencing

The four samples, not providing the same result for both methods, were tested using a previously published TaqMan based assay [10] and by fast COLD-PCR followed by sequencing. For sample ID 73 the TheraScreen kit was positive for the c.35 G > A mutation, and negative by CADMA. Sample ID 65 was positive for the c.34 G > A mutation and the c.35 G > C mutation by the TheraScreen kit, and only positive for the c.35 G > C mutation by CADMA. Sample ID 91 was positive for the c.35 G > A mutation by the TheraScreen kit and the c.38 G > A as well as the c.35 G > A mutation by CADMA. Finally, sample ID 96 was positive for the c.34 G > T mutation by the TheraScreen kit and the c.34 G > T and c.35 G > A mutations by CADMA. The CADMA results were confirmed by the TaqMan assay for all four samples. When using the COLD-PCR assay the CADMA and TaqMan results were confirmed for sample ID 65, 73, and 96. In sample ID 91, the c. 38 G > A mutation detected by CADMA and TaqMan was confirmed, however, the c.35 G > T mutation detected by CADMA, TaqMan, and the TheraScreen kit was not detected by COLD-PCR (Additional file 2: Table S1).

## Discussion

Screening for well-characterized point mutations predicting response or non-response to targeted therapies has in recent years become widely implemented in clinical settings. However, conventional PCR followed by Sanger sequencing, pyrosequencing, or HRM analysis may often fail to identify mutations in samples with a substantial wild-type DNA content [6,13,14]. It has also been shown that increased detection sensitivity may be used to identify additional patients which do not respond to anti-EGFR treatment [14]. For this reason, there is an increasing need for sensitive, reliable, and cost-effective methods for the detection of known mutations.

We have developed assays for detection of the seven most common *KRAS* mutations based on our recently developed method named Competitive Amplification of Differentially Melting Amplicons (CADMA), which may be combined with COLD-PCR to further increase the sensitivity of the assay [9]. However, clinical decision making based upon very low prevalence *KRAS* mutations is controversial, as it has not yet been clarified how mutation heterogeneity within CRC tumours affects outcome in patients treated with EGFR antibodies. Therefore, we decided not to combine the CADMA assays with COLD-PCR in the present study. Nevertheless, the analytical sensitivities of all assays were at least 0.5% mutant alleles in a wild-type background. These results were confirmed in another laboratory. However, it could be observed that the separation between the cell line dilutions and the wild-types were more pronounced in some runs than others, indicating that some in between run variation may occur. In between run variation is most likely to be caused by varying primer concentrations in the master mixes, which were prepared on a run to run basis, or variation caused by the instruments.

We did not assess the analytical sensitivity of the commercially available TheraScreen kit, which has been reported by the company to be sensitive to 1% mutant alleles in a wild-type background. However, it has been reported that diagnostic samples derived from FFPE tissues often may not be analyzed at this level of sensitivity [15]. Nevertheless, we have recently shown that the TheraScreen kit can detect mutations in samples derived from FFPE tissues, which could not be detected by conventional PCR followed by HRM or sequencing [6].

Here, we have analyzed mCRC samples from 100 patients using the TheraScreen kit and the CADMA assays. To facilitate direct comparison between the two methods, the samples were analyzed at 1% mutant level when using CADMA. Overall, consensus between the two methods was very high (95.9%), and in four out of four cases where different results were observed, the CADMA result could be confirmed by a previously published TaqMan based assay, which is sensitive to about 1% mutant alleles in a wild-type background [10], and by COLD-PCR followed by Sanger sequencing. However, in one sample a mutation detected by CADMA, TaqMan, and the Therascreen kit was not detected by COLD-PCR. Therefore, it is likely that the TheraScreen kit gave false positive results in two cases (c.34 G > A in sample ID 65 and c.35 G > A in samples ID 73) and false negative results in two cases (c.38 G > A in sample ID 91 and c.35 G > A in sample ID 96). Nevertheless, mCRC patients are *pro tem* only classified as mutation positive or negative for selection of treatment groups. Therefore, only one (sample ID 73) of the 100 patients studied is likely to have been misclassified. However, as previously mentioned, evidence is now present that patients harboring the codon 13 c.38 G > A mutation may benefit from anti-EGFR treatment [4,5,14]. False positive and negative results have previously been observed when using the TheraScreen kit in the order 1-2% [16].

We used 50 ng of DNA in the reactions, but it is likely that the CADMA assays will perform equally well using 25 ng or less DNA, which we have shown in a study of *BRAF* mutations in FFPE cutaneous malignant melanoma samples [13].

Many different methods have been developed with the goal of increasing the analytical sensitivity of mutation testing, however, increased sensitivity often comes with the cost of lower accuracy, increased complexity, and higher costs [17]. The TheraScreen kit uses a combination of mutation specific PCR primers and Scorpion probes, and is relatively expensive and labor-intense compared to most other methods employed for *KRAS* mutation detection [8]. CADMA uses HRM analysis to determine mutation status, which can be performed in a closed tube format without the use of any labeled oligonucleotides. HRM has been used for a range of different applications in molecular diagnostics due to its cost-effectiveness and convenience [18]. Compared to the TheraScreen kit and the TaqMan based assay, CADMA does not require a control assay as the real-time PCR data serves as a control for the quality of the sample, and thus the mutation status can be determined directly after a single run. However, scoring the samples of unknown mutation status is not always straightforward as visual inspection of both the derivative of the raw data (melt curve analysis) and the normalized HRM and difference graphs (high resolution melting analysis) may be needed for correct interpretation of the melting data.

The CADMA assays presented here have not been optimized to perform at a specific annealing temperature, which would have been an advantage as one sample then could be tested for all seven mutations in a single run. However, this may be achieved by changing the concentration of the overlapping primer and/or by designing new mutation specific primers. Incorporation of locked nucleic acids (LNAs) in the mutation specific primers may also make this task easier as the temperature window, where the mutation specific primer distinguish well between mutated and wild-type sequences, may be expanded. It may also be possible to multiplex CADMA assays while still being able to distinguish between different mutations, if one mutation specific primer introduces melting temperature decreasing mutations in the resulting mutated amplicon, and another mutation specific primer introduces melting temperature increasing mutations in the other resulting mutated amplicon.

The possibility to detect low or moderate abundance mutations is important in many different aspects of molecular diagnostics [19]. The mutation to be detected is often well characterized as is the case for *KRAS* mutations and other mutations in genes such as *BRAF*, *EGFR*, and *PIK3CA*. However, when the mutation to be detected with high sensitivity is unknown, other methods such as COLD-PCR [7] or Ice-COLD-PCR [20] followed by sequencing or pyrosequencing is recommended. Ice-COLD-PCR uses a synthetic wild-type-specific oligonucleotide reference sequence (RS), which is slightly shorter than the length of the PCR amplicon, so that it obstructs primer binding, and thereby inhibits amplification of wild-type alleles when using a five step PCR as described [20]. The RS contains a 3’-phosphate modification to prevent polymerase extension, and should be used together with a polymerase that lacks 5’- to 3’-exonuclease activity to prevent potential problems by its hydrolysis. The advantage of Ice-COLD-PCR is that the RS can be relatively long compared to the use of short wild-type-blocking oligonucleotides, which can be used without COLD-PCR [21], thereby allowing sensitive screening for unknown mutations of longer DNA sequences. Wild-type-blocking oligonucleotides which contain LNAs have been used to further increase the sensitivity of *BRAF* and *KRAS* mutation detection in Wild-type blocking PCR (WTB-PCR) [22,23]. Though WTB-PCR has proven to be highly sensitive, it is not performed in a closed-tube format, as sequencing of the PCR product is necessary.

## Conclusions

In conclusion, CADMA may improve *KRAS* mutation screening in mCRC. The use of an overlapping primer, which competes with the mutation specific primer for target binding may reduce or eliminate false amplification otherwise often observed in allele-specific PCR. In addition, the robust amplification of samples containing low abundance mutations provided by the overlapping primer may prevent false negative results. CADMA is performed in a closed tube format and mutation status can be determined directly by HRM analysis.

## Competing interests

Aarhus University has filed a patent application concerning the CADMA methodology with Kristensen LS, Hager H, and Hansen LL listed as inventors.

## Authors’ contributions

TEK carried out the molecular genetic studies, analysed the data, and co-wrote the manuscript. HH and LLH participated in the study design and co-wrote the manuscript. LSK conceived the study, participated in the study design, analysed the data, and co-wrote the manuscript. All authors read and approved the final manuscript.

## Pre-publication history

The pre-publication history for this paper can be accessed here:

http://www.biomedcentral.com/1471-2407/12/548/prepub

## Supplementary Material

Additional file 1 **Figure S1.** The analytical sensitivity and specificity of the CADMA assays performed using the Rotorgene Q. Ten wild-type replicates were run together with a standard dilution series of mutant alleles from cell lines carrying the relevant mutations in a wild-type background (50%, 10%, 1%, and 0.5%) in triplicates. The three replicates of the 0.5% standard could all be distinguished from ten wild-type replicates in all assays. A. The c.34 G > A CADMA assay. B. The c.38 G > A CADMA assay. C. The c.35 G > A CADMA assay. D. The c.34 G > C CADMA assay. E. The c.35 G > T CADMA assay. F. The c.34 G > T CADMA assay. Click here for file

Additional file 2 **Table S1.** Overview of the results from screening 100 mCRC samples using the TheraScreen kit and CADMA. A TaqMan based assay and COLD-PCR followed by sequencing were used to test samples for which the TheraScreen kit and CADMA did not give the same result. Click here for file
