# Supplementary figures and images for: Competitive amplification of differentially melting amplicons (CADMA) improves KRAS hotspot mutation testing in colorectal cancer
Source: BMC Cancer. 2012 Nov 23;12:548. doi: 10.1186/1471-2407-12-548 (PMC3517778; doi:10.1186/1471-2407-12-548)

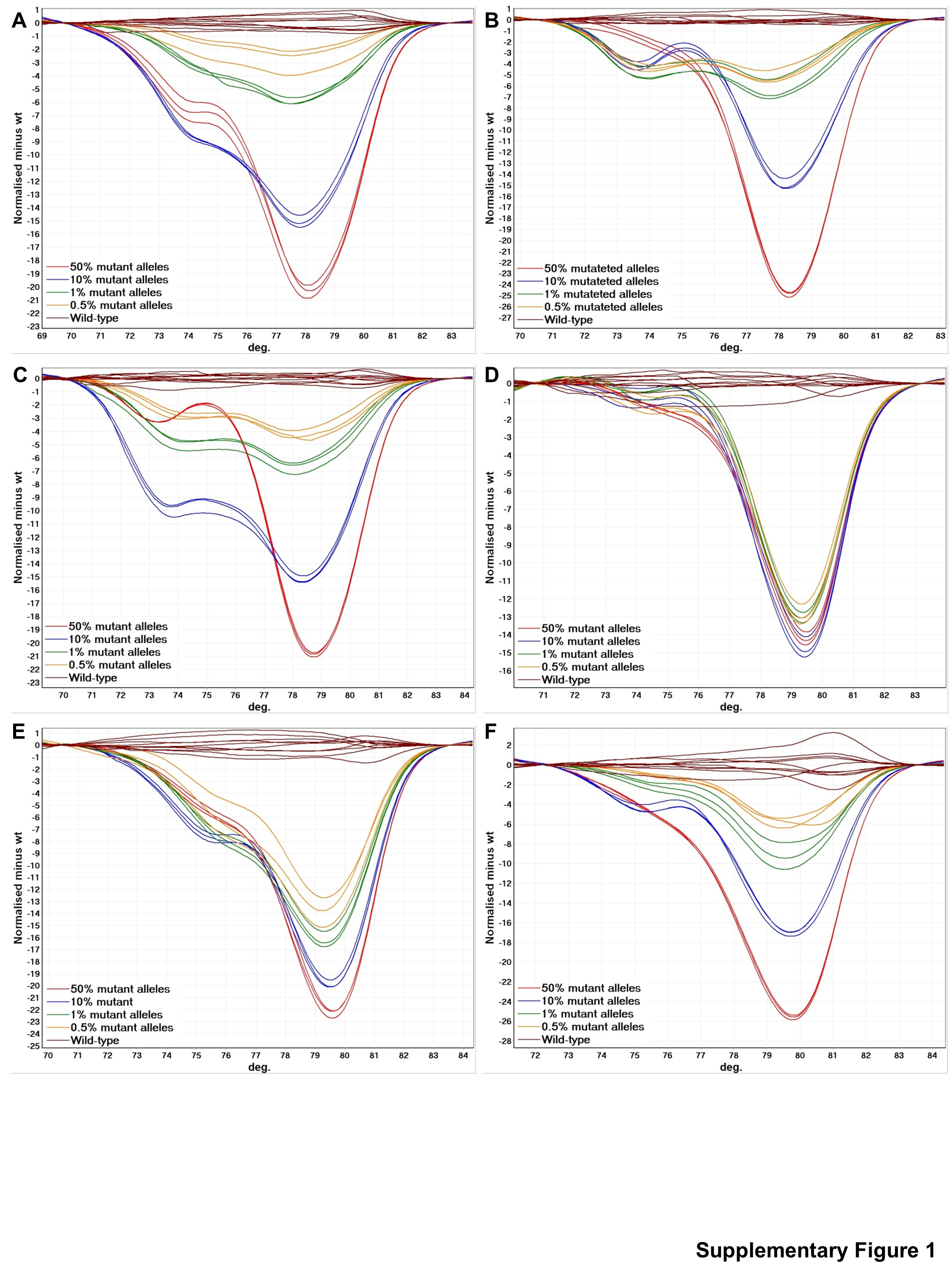

Supplement: Additional file 1 — Figure S1. The analytical sensitivity and specificity of the CADMA assays performed using the Rotorgene Q. Ten wild-type replicates were run together with a standard dilution series of mutant alleles from cell lines carrying the relevant mutations in a wild-type background (50%, 10%, 1%, and 0.5%) in triplicates. The three replicates of the 0.5% standard could all be distinguished from ten wild-type replicates in all assays. A. The c.34 G > A CADMA assay. B. The c.38 G > A CADMA assay. C. The c.35 G > A CADMA assay. D. The c.34 G > C CADMA assay. E. The c.35 G > T CADMA assay. F. The c.34 G > T CADMA assay. [file 1471-2407-12-548-S1.tiff]
